# Tumor Necrosis Factor Alpha -308G/A Gene Polymorphisms Combined with Neutrophil-to-Lymphocyte and Platelet-to-Lymphocyte Ratio Predicts the Efficacy and Safety of Anti-TNF-α Therapy in Patients with Ankylosing Spondylitis, Rheumatoid Arthritis, and Psoriasis Arthritis

**DOI:** 10.3389/fphar.2021.811719

**Published:** 2022-01-21

**Authors:** Ziran Wang, Lingjun Kong, Han Zhang, Fengchun Sun, Zijian Guo, Rui Zhang, Yaling Dou

**Affiliations:** ^1^ Department of Clinical Laboratory, Peking Union Medical College Hospital, Chinese Academy of Medical Sciences, Beijing, China; ^2^ Department of Clinical Laboratory, Affiliated Hospital of Jining Medical University, Jining, China

**Keywords:** Ankylosing spondylitis, rheumatoid arthritis, psoriasis arthritis, tumor necrosis factor alpha, neutrophil-to-lymphocyte ratio, platelet-to-lymphocyte ratio

## Abstract

**Background:** TNF-α has been reported to be closely associated with autoimmune inflammatory diseases. This study aims to investigate the role of *TNF-α* -308(rs1800629) G/A gene polymorphisms as well as neutrophil-to-lymphocyte ratio (NLR) and platelet-to-lymphocyte ratio (PLR) in predicting the efficacy and safety of TNF inhibitors (TNFi) in patients with ankylosing spondylitis (AS), rheumatoid arthritis (RA), and psoriasis arthritis (PsA).

**Methods:** A total of 515 subjects (181 AS, 144 RA, 48 PsA, 10 hyperbilirubinemia, 10 hyperlipidemia and 122 healthy control) were recruited in this study. The accuracy of RT-PCR methods for identifying individual *TNF-α* -308 genotypes was assessed using sequencing as the gold standard. Baseline NLR and PLR of patients with AS, RA and PsA and healthy controls (HC) were calculated and compared. Meanwhile, differences between responders and non-responders to TNFi treatment as well as between individuals with and without adverse effects (AE) among responders were compared.

**Results:** The RT-PCR method is stable and reliable for *TNF-α* -308G/A gene polymorphism analysis, independent of sample status. The GG genotype was overwhelmingly represented, with relatively few GA genotype, whilst the AA genotype was not detected in this study. There was no observed association between TNF-α-308G/A polymorphism and susceptibility in AS, RA or PsA patients. Patients with AS, RA, and PsA had a higher NLR, compared to the HC group. Apart from PsA patients, AS and RA patients had a higher PLR, compared to the HC group. NLR was positively correlated with PLR. Furthermore, a lack of response was more frequently observed in AS and RA patients that carrying the GA genotype than the GG genotype. AS and RA patients with AE had higher NLR and PLR, compared with the non-AE group.

**Conclusion:** Our study preliminarily shown that combining *TNF-α* -308G/A polymorphisms with NLR and PLR can predict the responsiveness and safety of anti-TNF therapy in patients with AS or RA.

## Introduction

Ankylosing spondylitis (AS), rheumatoid arthritis (RA) and psoriasis arthritis (PsA) are common autoimmune-mediated inflammatory diseases. The primary clinical feature of AS is the involvement of peripheral joints, which eventually leads to ossification of entheses and loss of joint mobility (Sode et al., 2018). According to a preliminary survey, the prevalence of AS in China is approximately 0.3% (Shen et al., 2013). AS can affect the quality of life, mortality and work capacity of patients, also imposes a heavy burden on society (Boonen and van der Linden, 2006). Simultaneously, RA is characterized by persistent synovitis, systemic inflammation, and autoantibodies. Individuals with RA have increased mortality due to cardiovascular disease and infection (Scott et al., 2010). PsA is an immune-mediated, genetic disease manifesting in the joints. Patients with psoriasis were at an increased risk of developing serious health problems, such as psoriatic arthritis, metabolic syndrome and cardiovascular disorders (Boehncke and Schön, 2015). Notably, it is difficult to achieve stable disease in PsA patients with concomitant other diseases. It has been reported that PsA patients with hepatic steatosis (HS) and carotid plaques (CPs) were at increased risk of relapse and failure to achieve minimal disease activity (MDA) after anti-TNF-α treatment (Di Minno et al., 2012). AS, RA, and PsA caused serious threats to human health, while both genetic and environmental factors play an important role in their pathogenesis.

Tumor necrosis factor alpha (TNF-α) is a cytokine that produced by macrophages or monocytes in acute inflammation which involved in a range of cellular signaling events (Idriss and Naismith, 2000). Serum levels of TNF-α have been reported to be significantly higher in patients with AS, RA, and PsA compared to the healthy individuals (Gratacós et al., 1994; Kyriakou et al., 2014). This implies that TNF-α may have an essential role in the development of autoimmune inflammatory diseases. On the other hand, single nucleotide gene polymorphisms (SNPs) of the *TNF-α* promoter are gradually being of interest. The G-to-A transition at position -308 (rs1800629) has been the most extensively investigated. However, the association of SNP at the *TNF-α* -308G/A with autoimmune diseases such as AS, RA, and PsA has not been fully elucidated.

In recent years, there is growing evidence of the value of neutrophil-to-lymphocyte ratio (NLR) and platelet-to-lymphocyte ratio (PLR) in assessing disease activity and prognosis in systemic inflammatory diseases (Qin et al., 2016; Wu et al., 2016; Gasparyan et al., 2019). Besides, elevated NLR and PLR are considered as biomarkers of poor prognosis in patients with cancer (Hirahara et al., 2019; Ni et al., 2020). Thus, NLR and PLR are inexpensive and easily accessible indicators that have a wide range of clinical applications. Unfortunately, studies of NLR and PLR in evaluating the efficacy and safety of anti-TNF-α therapy in patients with AS, RA, and PsA are still lacking.

Resistance to conventional drugs has become a major challenge in the treatment of autoimmune diseases. Based on the hypothesis that excessive TNF-α accumulation may cause deleterious effects, a variety of treatments targeting TNF-α (such as Infliximab, Etanercept, Certolizumab pegol, Adalimumab) have been introduced in autoimmune diseases and have shown favorable performance (Chatzantoni and Mouzaki, 2006). However, 40–60% of patients did not respond to TNF-α inhibitors (TNFi) while they were exposed to the risk of serious adverse effects, including serious infections and even oncogenesis (Strand et al., 2007). Biomarkers based on individual differences are desirable to evaluate the effectiveness of anti-TNF-α therapy. Several studies have shown that *TNF-α* -308G/A can be an excellent predictor of the responsiveness to TNF-α inhibitors (Maxwell et al., 2008; Netz et al., 2017). Similarly, NLR and PLR have been preliminarily studied in predicting the responsiveness and drug persistence of anti-TNF-α agents in patients with RA (Lee et al., 2019). Specifically, their predictive power in AS, RA, and PsA needs to be further investigated.

In this study, we focused on the utility of *TNF-α* -308G/A polymorphisms, NLR, and PLR as potential biomarkers in AS, RA, and PsA, as well as the role of *TNF-α* -308G/A polymorphisms in combination with NLR and PLR in predicting the efficacy and safety of anti-TNF-α therapy.

## Materials and Methods

### Subjects

A total of 515 subjects (181 AS, 144 RA, 48 PsA, 10 hyperbilirubinemia, 10 hyperlipidemia and 122 healthy control) were recruited from Peking Union Medical College Hospital (PUMCH), Chinese Academy of Medical Sciences between July 2019 and August 2021 in this study. Of these patients, 181 patients were diagnosed with AS according to the Modified New York criteria by the American College of Rheumatology (van der Linden et al., 1984). 144 patients were defined as RA according to the American Rheumatism Association 1987 revised criteria (Arnett et al., 1988) and 48 patients were defined as PsA fulfilling classification criteria for psoriatic arthritis (Taylor et al., 2006). Hyperbilirubinemia (HB) and hyperlipidemia (HLP) were diagnosed by combining clinical symptoms and laboratory results (Sullivan and Rockey, 2017; Stewart et al., 2020). Data including demographic information, medical history, laboratory results and drug consumption information were collected through the hospital information system (HIS) and laboratory information system (LIS) of PUMCH. The eligibility criteria of patient-recruitment were: (1) confirmed diagnosis of RA, AS, PsA, HB, or HLP; (2) complete demographic data, medical history can be obtained; (3) EDTA anticoagulated whole blood samples with acceptable quality were available. The exclusion criteria adopted in the research were as follows: (1) presence of other autoimmune diseases, malignancies, acute infections, hematological diseases, immunodeficiency diseases and other serious conditions; (2) exposure to biologic therapy due to other diseases prior to enrollment; (3) patients have been misdiagnosed several times and have received several inappropriate treatments before being properly diagnosed; (4) an unwillingness or inability to cooperate. In addition, the healthy individuals selected as control group had no family history of autoimmunity or chronic inflammatory disease and no abnormal laboratory results. Duplicate individuals were removed using a unique identification code while the whole blood collected was kept with EDTA from individuals for subsequent experiments. The NLR is calculated dividing the absolute count of neutrophils by the absolute count of lymphocytes, and the PLR is calculated dividing the platelet count by the absolute count of lymphocytes. The patient’s responsiveness to TNF-α inhibitors was evaluated by the clinicians based on a combination of the patient’s joint or spinal involvement, patient description and laboratory testing. For AS, patients were defined as responders if their Ankylosing Spondylitis Disease Activity Score (ASDAS) decreased by ≥ 1.1 units compared to baseline score, otherwise they were defined as non-responders (Machado et al., 2011). Disease Activity Score using 28 joint counts (DAS28) was used to assess the responsiveness of patients with RA. Patients with DAS28 reduction >1.2 or DAS28 < 5.1 and a DAS28 reduction between 0.6 and 1.2 were considered responders, otherwise they were considered non-responders (van Riel and Renskers, 2016). For PsA, the treatment response was evaluated by the Disease Activity Index for Psoriatic Arthritis (DAPSA). Patients were regarded as responders when their DAPSA score improved by 50% or more relative to baseline levels, otherwise they were regarded as non-responders (Schoels et al., 2016).

### Ethics Statement

This study was conducted in accordance with the recommendations of the PUMCH. All procedures performed in studies involving human participants were in accordance with the ethical standards of the institutional and/or national research committee with the 1964 Helsinki declaration and its later amendments or comparable ethical standards. This study was approved by the Ethics Committee of PUMCH (No. HS2019035).

### Genotyping

Genomic DNA was extracted from whole peripheral blood via DNA extraction kits (Tianlong Technology Co. LTD, Xi’an, China). Genotyping for *TNF-α* -308 (rs1800629) was performed using *TNF-α* -308G/A gene polymorphisms RT-PCR detection kit (Wuhan HealthCare Biotechnology Co., Ltd., Wuhan, China). Genotypes were interpreted with the criteria of Supplementary Table S1. Meanwhile, genotyping was achieved by TaqMan chemistry using the Applied Biosystems real-time Prism 3730XL Sequence Detection System (ABI Inc. CA, United States) according to the Applied Biosystem protocol.

### Statistical Analysis

All the data were analyzed by Excel 2019 (Microsoft Inc., United States), and SPSS 20.0 (SPSS Inc., Chicago, IL, United States) software. The concordance between genotyping methods was analyzed by using kappa test. Correlation tests were performed by using the Spearman’s correlation analysis. Differences between groups were assessed using the Wilcoxon or Chi-square test. The odds ratios (ORs) and 95% confidence intervals (CIs) were calculated by using logistic regression analysis. The Hardy-Weinberg equilibrium (HWE) was evaluated by online website (http://ihg.gsf.de/cgi-bin/hw/hwa1.pl). *p* < 0.05 was considered statistically significant.

## Results

### Methodological Assessment of Genotype Identification

**FIGURE 1:**
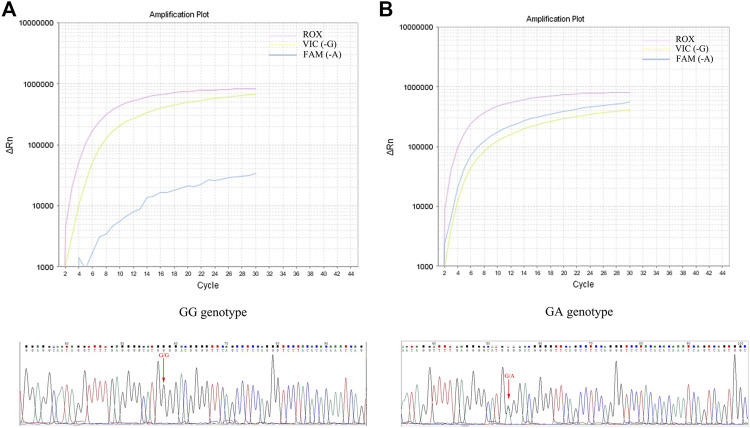
Determination of *TNF-α* -308G/A genotype using RT-PCR and sequencing. **(A)**: GG genotype amplification curve and sequencing peaks map; **(B)**: GA genotype amplification curve and sequencing peaks map.

In this study, the *TNF-α* -308G/A polymorphisms detection kit (RT-PCR) was used to identify the genotype of each subject. To ensure the reliability of genotype identification for each participant, a subset of subjects (60 HC, 172 AS, 123 RA, 37 PsA, 10 HB and 10 HLP) were selected to assess the accuracy of the RT-PCR method, using the sequencing method as the gold standard. As depicted in Table 1; Figure 1, there was an excellent agreement between the two methods in the HC, AS, RA and PsA populations. Notably, favorable results were also achieved with the RT-PCR method in patients with HB and HP, indicating that RT-PCR method is accurate and reliable, independent of bilirubin and lipid interference in the samples.

**TABLE 1:** Detection of the genotype of the *TNF-α* -308G/A by RT-PCR and sequencing.

Group	Sample size	RT-PCR			Sequencing			Kappa value	*P* value
		GG	GA	AA	GG	GA	AA		
HC	60	55	5	0	55	5	0	1	<0.001
AS	172	162	10	0	162	10	0	1	<0.001
RA	123	118	5	0	118	5	0	1	<0.001
PsA	37	36	1	0	36	1	0	1	<0.001
HB	10	10	0	0	10	0	0	1	<0.001
HLP	10	10	0	0	10	0	0	1	<0.001

AS, Ankylosing Spondylitis; HB, hyperbilirubinemia; HC, Healthy Control; HLP, hyperlipidemia; PsA, Psoriatic Arthritis; RA, Rheumatoid Arthritis.

### Genotype Distributions of *TNF-α* -308G/A Gene Polymorphisms in the RA, AS, PsA Patients and HC

A total of 181 patients with AS, 144 with RA, 48 with PsA and 122 HC were included in the study to examine the polymorphisms of *TNF-α* -308G/A. The clinical characteristics and baseline laboratory data of all participants were summarized in Table 2. All samples were genotyped by RT-PCR method. The distribution and frequency of *TNF-α* -308G/A genotypes were shown in Table 3. The genotypes distribution agreed with the Hardy-Weinberg equilibrium (*p* = 0.52). Overall, the GG genotype was overwhelmingly represented, with relatively few GA genotype, whilst the AA genotype was not detected in this study. Furthermore, logistic regression analyses revealed that *TNF-α* -308G/A polymorphisms were not associate with susceptibility of AS, RA, or PsA (*p* > 0.05).

**TABLE 2:** Clinical characteristics and baseline laboratory data in HC and patients with AS, RA and PsA.

Variable	HC (*n* = 122)	AS (*n* = 181)	RA (*n* = 144)	PsA (*n* = 48)
Age, years, median (IQR)	42 (33–52)	35 (29–45)	51 (40–60)	46.5 (34–53)
Sex, Male/Female	44/78	131/50	24/120	24/24
CRP, mg/L, median (IQR)	−	11.82 (3.87–23.49)	4.99 (1.51–26.29)	5.67 (2.10–16.47)
ESR, mm, median (IQR)	−	18 (7–34)	23 (12–51)	14 (6.5–27.5)
NLR, median (IQR)	2.01 (1.62–2.30)	2.25 (1.81–2.91)	2.40 (1.80–3.11)	2.49 (1.58–3.26)
PLR, median (IQR)	134.6 (111.7–163.4)	147.2 (117.5–180.2)	152.4 (118.2–203.7)	137.2 (100.7–189.9)
HLA-B27 positive/negative	−	97/22	−	−
RF, U/L, median (IQR)		−	91 (24.9–228)	−
Anti-Anti-CCP antibody, positive/negative	−	−	86/31	−
Treatment, n				
NSAIDs	−	55	63	15
DMARDs	−	45	97	5
GC	−	10	37	5
TCM	−	10	5	5
Dermatological drug	−	−	−	14
Anti-TNF-α agents	−	97	28	22
Others	−	4	31	7

AS, Ankylosing Spondylitis; CCP, cyclic citrullinated peptide; CRP, C-reactive protein; DMARDs, disease modifying antirheumatic drug; ESR, erythrocyte sedimentation rate; GC, glucocorticoids; HC, Healthy Control; IQR, interquartile range; NLR, neutrophil-to-lymphocyte ratio; NSAIDs, nonsteroidal anti-inflammatory drugs; PLR, platelet-to-lymphocyte ratio; PsA, Psoriatic Arthritis; RA, Rheumatoid Arthritis; RF, rheumatoid factor; TCM, traditional Chinese medicine.

**TABLE 3:** Genotype frequencies of *TNF-α* -308G/A in patients with AS, RA, PsA and HC.

Gene	SNP	Group	Sample size	Genotype			OR (95%CI)	*P* value
				GG	GA	AA		
*TNF-α*	rs1800629 (-308 G>A)	HC	122	114 (93.44%)	8(6.56%)	0(0.00%)	Reference	
		AS	181	171(94.48%)	10(5.52%)	0(0.00%)	0.83 (0.31–2.14)	0.81
		RA	144	135(93.75%)	9(6.25%)	0(0.00%)	0.95 (0.38–2.52)	1.00
		PsA	48	47(97.92%)	1(2.08%)	0(0.00%)	0.30 (0.03–2.10)	0.45

AS, Ankylosing Spondylitis; HC, Health Control; OR, Odds Ratio; PsA, Psoriatic Arthritis; RA, Rheumatoid Arthritis.

### NLR and PLR in the RA, AS, PsA Patients and HC

**FIGURE 2:**
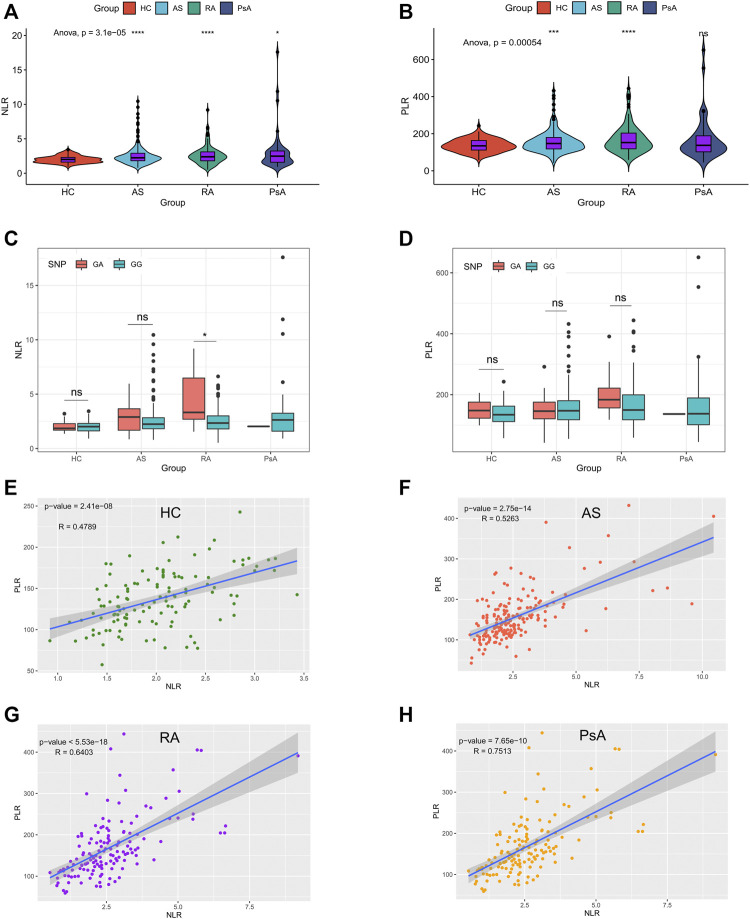
NLR and PLR in the RA, AS, PsA patients and HC. **(A–B)**: baseline levels of NLR and PLR in subjects in the AS, RA, PsA, and HC groups, ****p < 0.0001, ***p < 0.001, *p < 0.05; **(C–D)**: baseline levels of NLR and PLR in subjects with different genotypes in AS, RA, PsA, and HC groups, *p < 0.05, ns, no significance; **(E–H)**: The correlation between PLR and NLR in HC, AS, RA, and PsA group.

To explore the potential value of NLR and PLR as biomarkers, baseline NLR and PLR of AS, RA, PsA patients and HC were calculated. As shown in Figure 2A, patients with AS, RA and PsA had a higher NLR, compared to the HC group. Apart from PsA patients, AS and RA patients had a higher PLR, compared to the HC group. (Figure 2B **)**. We further compared the differences of NLR and PLR among individuals with different genotypes and showed that only in the RA group, patients with the GA genotype had a higher NLR than the GG genotype. (Figures 2C,D **)**. It was intriguing to note that NLR was positively correlated with PLR in the AS (r = 0.53, *p* < 0.001), RA (r = 0.64, *p* < 0.001), PsA (r = 0.75, *p* < 0.001) and HC groups (r = 0.48, *p* < 0.001). (Figures 2E–H **)**. Moreover, we observed a positive correlation among NLR, PLR, CRP and ESR in the AS and RA groups. (Supplementary Figure S1).

### *TNF-α* -308G/A Polymorphisms Combined With Baseline NLR and PLR Predict Responsiveness and Safety of TNF-α Inhibitors

In this study, we collected information on medication administration for all patients *via* the HIS system. As illustrated in Table 2, non-steroidal anti-inflammatory drugs (NSAIDs) and disease modifying antirheumatic drug (DMARDs) were often the first choice of drugs for the treatment of auto-immune inflammatory diseases. Unfortunately, we have observed that a significant proportion of patients do not respond to these traditional medicines. The introduction of TNFi brought a ray of hope for these patients. Anti-TNF-α therapies are now widely used in the clinic, however, there are still some patients who are not responsive to TNFi and under enormous financial pressure as well as being exposed to the risk of adverse effects (AE).

In this study, the AS, RA and PsA groups had 97, 28 and 22 patients who received TNFi such as etanercept or adalimumab. (Table 4). Most patients showed satisfactory efficacy with TNFi, but a few non-responders remained. The differences in clinical features between responders and non-responders in patients with AS and RA have been shown in Table 5. We found a lack of response was more frequently observed in AS and RA patients carrying the GA genotype than the GG genotype (*p* < 0.05). For RA patients, non-responders had higher level of NLR and PLR compared to responders, whereas for AS patients this difference was not observed. Therefore, *TNF-α* -308G/A polymorphisms may be used as a predictor of responsiveness to TNFi.

**TABLE 4:** Categories and therapeutic effectiveness of TNF-α inhibitors in patients with AS, RA and PsA.

Treatment information	AS	RA	PsA
TNF-α inhibitors	97	28	22
Etanercept	63	17	8
Adalimumab	11	6	4
Certolizumab pegol	1	0	5
Golimumab	4	0	0
Infliximab	0	1	0
2 TNFi^a^	17	4	5
3 TNFi^a^	1	0	0
Treatment outcome	97	28	22
Responders	82	21	18
Non-Responders	5	3	2
Others^b^	10	4	2
Adverse effects in GG genotype responders	27	9	6
Somatic pains	16	9	4
Iritis or tuberculosis	9	0	2
Lymphadenectasis	1	0	0
Liver damage	1	0	0

a Switching to the 2nd or 3rd TNFi due to disease progression or adverse effects.

b Follow-up time of less than 3 months.

AS, Ankylosing Spondylitis; PsA, Psoriatic Arthritis; RA, Rheumatoid Arthritis; TNFi, TNF-α, inhibitors.

**TABLE 5:** Differences in clinical features between responders and non-responders in patients with AS and RA.

Predictors	AS			RA		
	Responders	Non-Responders	*P* value	Responders	Non-Responders	*P* value
Sex (Male vs Female)	57/25	5/0	0.32	3/18	3/0	1.00
Age, years, median (IQR)	33 (28–42)	33 (26–46.5)	0.92	49.33 ± 14.96	41.67 ± 11.5	0.41
CRP, mg/L, median (IQR)	13.35 (4.67–29.76)	13.73 (3.97–18.13)	0.64	3.74 (1.62–28.13)	62.05 (1.69–85.93)	0.31
ESR, mm, median (IQR)	21 (8–41.25)	11 (2.5–41.5)	0.19	20 (10–52)	87 (13–88)	0.21
NLR, median (IQR)	2.18 (1.71–2.73)	3.06 (1.52–3.47)	0.41	2.26 (1.43–2.94)	6.48 (2.94–6.66)	**0.02**
PLR, median (IQR)	141.9 (118.8–167.2)	152.2 (92.6–164.9)	0.97	141.3 (106.4–186.1)	221.5 (204.6–277.0)	**0.02**
TNF-α gene (-308) rs1800629 (GA vs GG)	0/82	3/2	**< 0.001**	1/20	2/1	**0.03**

AS, Ankylosing Spondylitis; CRP, C-reactive protein; ESR, erythrocyte sedimentation rate; IQR, interquartile range; NLR, neutrophil-to-lymphocyte ratio; PLR, platelet-to-lymphocyte ratio; RA, Rheumatoid Arthritis. The results are in bold if *P* < 0.05.

**FIGURE 3:**
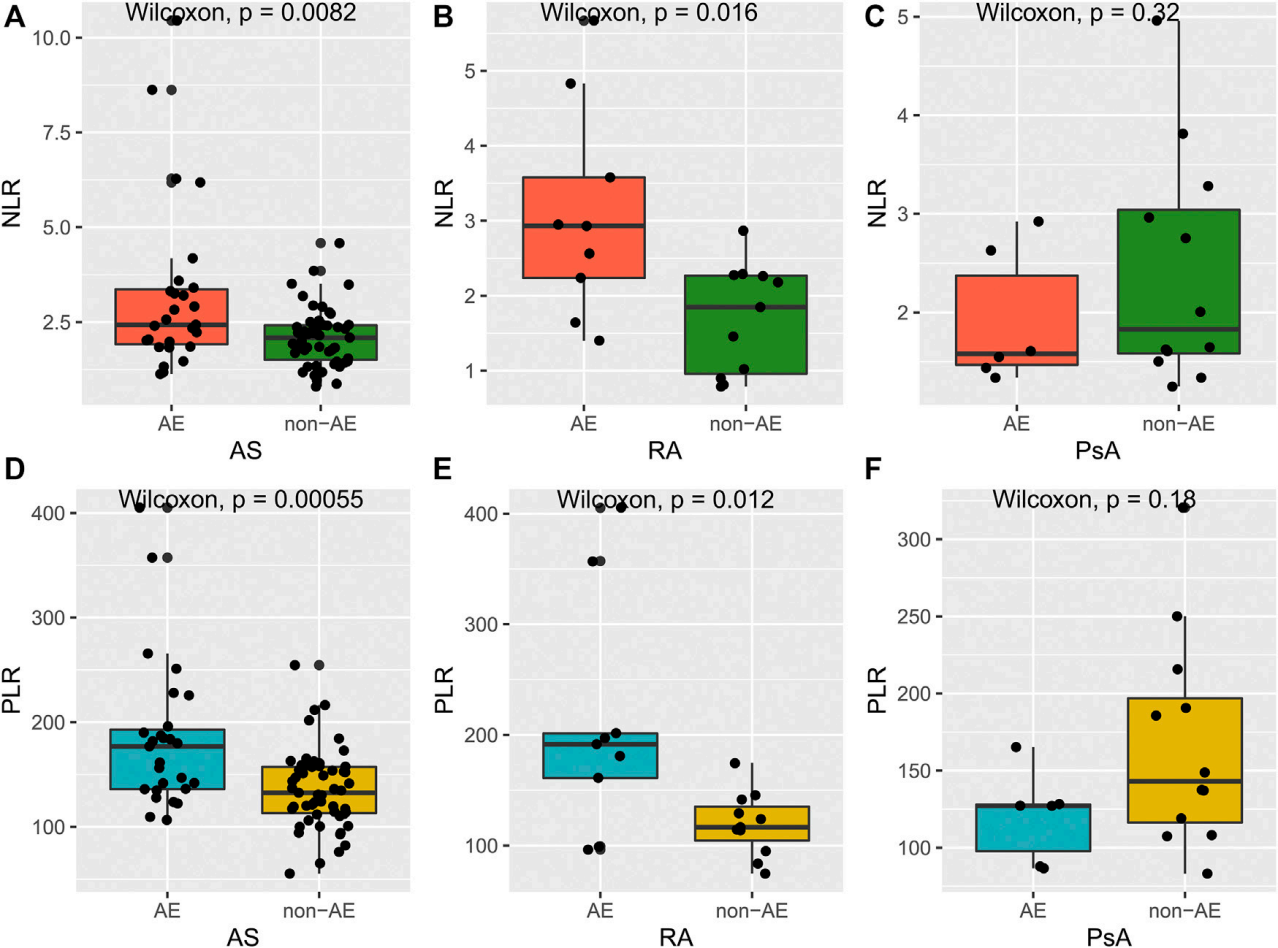
Differences of NLR and PLR between AE responders and non-AE responders. **(A–C)**: differences of NLR between AE responders and non-AE responders in AS, RA, and PsA group; **(D–F)**: differences of PLR between AE responders and non-AE responders in AS, RA, and PsA group.

**FIGURE 4:**
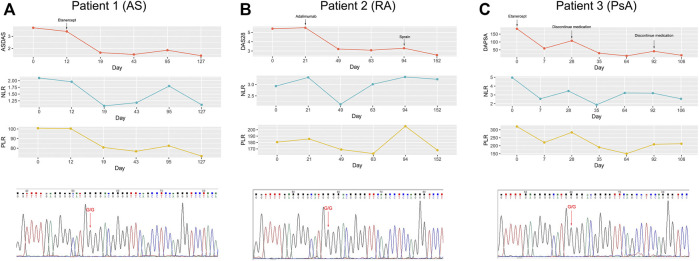
Correlation of changes in disease activity with changes in PLR and NLR over the course of TNFi treatment. **(A)**: patient 1 with AS carrying the GG genotype; **(B)**: patient 2 with RA carrying the GG genotype; **(C)**: patient 3 with PsA carrying the GG genotype.

Remarkably, we also observed that most patients carrying the GG genotype showed a superior response to TNFi, but there were still some patients suffering from AE, such as somatic pains, iritis, or tuberculosis, which led them to switch to another TNFi or even abandon anti-TNF-α treatment. Meanwhile, patients carrying the GG genotype have a wide range of values for NLR and PLR. On this basis, we intended to explore whether NLR and PLR could further identify patients exposed to the risk of AE. As shown in Figure 3, AS and RA patients with AE had higher NLR and PLR, compared with the non-AE group. However, for PsA patients, there was no significant difference in NLR and PLR between the AE and non-AE groups. In addition, we also found that during the process of anti-TNF-α treatment, the change trend in NLR, PLR over time was consistent with the change in disease activity (Figure 4). Overall, combining *TNF-α* -308G/A polymorphisms with NLR and PLR can predict the responsiveness and safety of anti-TNF therapy in patients with AS or RA.

## Discussion

TNF-α was initially identified as a factor that can induce tumor cell necrosis, but was latterly found to be involved in the pathogenic process of autoimmune diseases as an important pro-inflammatory factor (Jang et al., 2021). The accumulation of TNF-α can ultimately contribute to the development of chronic inflammation and tissue destruction. It has been reported that *TNF-α* promoter -308A allele in Crohn’s disease increased TNF production promoting inflammatory activity and were associated with worse responsiveness to anti-TNF-α therapy (González et al., 2003; Netz et al., 2017). On this basis, we speculated that *TNF-α* -308A may also have a similar role in autoimmune diseases such as AS, RA, and PsA.

Various methods have been reported to determine the genotype of *TNF-α* -308G/A, such as matrix-assisted laser desorption/ionization time-of-flight mass spectrometry (MALDI-TOF MS) (Sun et al., 2013), restriction fragment length polymorphism (RFLP) (Manolova et al., 2014), and high resolution melting (HRM) (Li et al., 2014). However, the accuracy of these methods as genotyping has rarely been verified. In this study, to identify each subject’s genotype accurately, we selected a subset of the population was genotyped using RT-PCR with the sequencing method as the gold standard. The results showed an extremely high level of agreement between the two methods. Besides, the genotype analysis of patients with HB and HLP was not interfered by bilirubin and lipids. Therefore, the RT-PCR method is stable and reliable for *TNF-α* -308G/A gene polymorphism analysis, independent of sample status.

Interestingly, the GG genotype was overwhelming (over 90%) in both disease groups and healthy controls, the GA genotype was relatively rare (2.08–6.56%), and the AA genotype was not detected. The frequency of genotype distribution in our study was analogous to two other studies from China (Sun et al., 2013; Hu et al., 2018). In contrast to our study, the frequency of the GA genotype was slightly higher in the Bulgarian and Danish populations, accounting for about 20% of the population (Manolova et al., 2014; Sode et al., 2018). In the UK population, the frequency of the GA genotype even reaches 32% (Maxwell et al., 2008). This discrepancy may be due to racial differences and suggested that the A allele in the *TNF-α* -308 gene is relatively scarce in the Chinese population.

The association between *TNF-α* -308 G/A and AS has been well studied but still controversial. Sode et al. and Hu et al. have reported that heterozygous variant genotypes of *TNF-α* -308G/A were associated with reduced risk of AS (Hu et al., 2018; Sode et al., 2018). Nevertheless, in the reports of Fraile et al. and Sun et al., no significant differences in the genotype and allele frequencies of *TNF-α* -308G/A (rs1800629) polymorphism were observed between AS patients and healthy individuals (Fraile et al., 1998; Sun et al., 2013). Our findings were analogous to the latter, with no explicit evidence of an association between AS and *TNF-α* -308G/A being observed. A meta-analysis based on 35 case-control studies showed that rs1800629 polymorphism significantly increased the risk of AS in Caucasians and decreased the risk of AS in mixed populations (Gao et al., 2021). Therefore, it is conceivable that race and sample size are crucial factors contributing to the conclusion and that future large-scale multicenter studies are needed to elucidate the role of *TNF-α* -308G/A polymorphisms in AS.

Our study also indicated that *TNF-α* -308G/A polymorphisms were not associated with susceptibility to RA. Li et al. reported that the rs1800629 A allele of *TNF-α* was negatively associated with RA in the Chinese population (Li et al., 2014). But several studies have concluded that *TNF-α* -308G/A polymorphisms were not associated with RA susceptibility (Sun et al., 2013; Manolova et al., 2014; Cadena-Sandoval et al., 2018). A meta-analysis of 19 studies involving 2584 patients with RA and 3254 controls revealed that the *TNF-α* rs1800629 G/A polymorphism may represent a significant risk factor for RA in Latin Americans but not in the European, Arab, or Asian populations (Song et al., 2014). It was consistent with our research.

For PsA, our study demonstrated that individuals with the GA genotype appear to have a reduced risk of PsA, but the difference was not statistically significant (OR = 0.30, 95%CI 0.03–2.10, *p* = 0.45). A previous meta-analysis consisting of 2253 psoriasis patients and 1947 controls highlighted that *TNF-α* -308G/A polymorphism was significantly associated with decreased risk of psoriasis (GA versus GG: OR 0.67, 95%CI 0.57–0.78, *p* < 0.001) (Zhuang et al., 2013). Perhaps the small sample size could explain the failure to observe significant differences in our results. Therefore, in the future, studies with larger samples containing different stages of PsA need to be performed to draw a reliable conclusion.

Generally, the neutrophil count in the blood increases as the inflammatory disease progresses, while the lymphocyte count decreases. There is growing evidence that platelets are activated and play vital roles in the inflammatory response (Thomas and Storey, 2015). Thus, NLR and PLR are regarded as robust indicators that reflect the progression of inflammatory diseases. Our study showed that patients with AS and RA had higher NLR and PLR compared with HC group, which is in accordance with previous reports (Erre et al., 2019; Zeb et al., 2019). Notably, NLR was elevated in PsA patients compared with HC group, while PLR was not. Surprisingly, our results were perfectly concordant with the study by Kim et al. (Kim et al., 2016). Mean platelet volume (MPV) has been reported to significantly increase in patients with PsA as a marker of platelet activation (Canpolat et al., 2010). Thus, both the number and volume of platelets may expand over the course of PsA whereas PLR solely considers the number of platelets. Furthermore, when we stratified the different disease groups and healthy controls by genotype, there were no significant differences regarding NLR and PLR between individuals carrying the GG genotype and the GA genotype. It implied that there was no obvious correlation between *TNF-α* -308G/A polymorphisms and NLR or PLR. Nevertheless, NLR was positively correlated with PLR in AS, RA, PsA patients and in the HC group, revealing a well-defined agreement between the NLR and PLR. When considering the association between NLR, PLR and other laboratory indicators, such as CRP or ESR, higher correlations were observed in patients with AS and RA. These correlations have also been confirmed in previous publications (Kim et al., 2016; Qin et al., 2016), suggesting NLR and PLR as new inexpensive and available biomarkers that can effectively reflect disease activity.

TNFi can be of significant benefit to patients with AS, especially in cases when conventional treatment has failed, but non-response to its application also raised a concern. On the other hand, TNFi is enormously expensive and carries the risk of adverse effects, thus there is an urgent need for a program to guide the use of the drug based on individual variation. Our survey data showed that 97 patients with AS, 28 patients with RA, and 22 patients with PsA were given treatment with TNFi and most of them exhibited a favorable outcome, while just ten were non-responders. Despite the small size of the non-response cases, the fact that six of the ten cases were of the GA genotype implied that the GA genotype may be a predictor of treatment failure. Our study agreed with the findings of Ma et al., in their study, the GG genotype was more frequent in good responders to TNF-α inhibitors in patients with AS (Ma et al., 2017). Certainly, given the rarity of the A allele in the Chinese population, continuous studies with larger populations are desirable to further clarify the relationship between *TNF-α* -308G/A polymorphisms and responsiveness to TNF-α inhibitors.

The association between *TNF-α* -308G/A polymorphism and responsiveness of TNFi in patients with RA was as well controversial. A meta-analysis conducted by Lee et al. asserted that RA patients carrying the A allele had a worse response to TNF-α inhibitors than those carrying the G allele, but they denied this in a consequent updated meta-analysis (Lee et al., 2006; Lee et al., 2010). Therefore, more and more rigorous studies will be needed in the future to reach a sufficiently scientific conclusion. Song et al. have reported that PsA individuals carrying the *TNF-α* -308 G allele shown better responsiveness to TNFi than those with A alleles in Caucasians (Song et al., 2015). Unfortunately, similar studies in Chinese populations are conspicuously rare. In our report, although the number of cases was limited, 18 of the 19 patients carrying the GG genotype responded to TNFi whilst one patient carrying the GA genotype did not respond. It was also an implicit association of *TNF-α* -308G/A polymorphisms with responsiveness to TNFi in Chinese PsA patients, which of course needs further verification subsequently.

Another concern about TNFi is its adverse effects. We have observed that some responders suffered from AE that forced them to change to another TNFi or to give up biological treatments. This situation imposed a heavy financial burden on them and taken a psychological toll on them. Lee et al. reported that in patients with RA, higher baseline NLR predicted poorer persistence of TNFi, including lack of efficacy and adverse events (Lee et al., 2019). Since NLR and PLR are taken in a wide range of values in responders, we speculated that NLR and PLR may be biomarkers for identifying patients at high risk of AE. We analyzed data on NLR and PLR in responders carrying the GG genotype and showed that in patients with AS and RA, NLR and PLR were higher in the AE group than in the non-AE group. This implied that responders exposed to AE may have a greater inflammatory burden that impaired the persistence of TNFi. In contrast, this difference was not observed in PsA patients. We considered two reasons might help explain it, one being that our study included fewer patients with PsA and the other being that because PsA has both psoriasis and arthritis symptoms, diagnosis is complicated and various treatments may have led to inflammatory storms in the body before a definitive diagnosis was obtained. Finally, we analyzed follow-up data on NLR and PLR during TNFi treatment and the results revealed a visible correlation between disease activity and NLR or PLR. Hence, NLR and PLR may be reliable biomarkers for the follow-up of patients undergoing TNFi treatment.

However, there were some limitations in our study. Firstly, our sample size was limited, and larger sample sizes would be required in future studies. Secondly, the A allele was remarkably scarce in our study in the Chinese population, but this was consistent with the relevant published studies across other cohorts. Thirdly, in this study we only examined the *TNF-α* -308G/A polymorphism; other SNPs of *TNF-α* should be included in future studies.

## Conclusion

Our study preliminarily shown that *TNF-α* -308G/A polymorphisms may be used as a potential biological marker to evaluate the responsiveness to TNFi in AS or RA patients. Higher baseline NLR and PLR could identify responders at higher risk of AE. Overall, combining *TNF-α* -308G/A polymorphisms with NLR and PLR can predict the responsiveness and safety of anti-TNF therapy in patients with AS or RA.

## Data Availability

The original contributions presented in the study are included in the article/Supplementary Materials, further inquiries can be directed to the corresponding author.
