# Supplementary material for: Tumor Necrosis Factor Alpha -308G/A Gene Polymorphisms Combined with Neutrophil-to-Lymphocyte and Platelet-to-Lymphocyte Ratio Predicts the Efficacy and Safety of Anti-TNF-α Therapy in Patients with Ankylosing Spondylitis, Rheumatoid Arthritis, and Psoriasis Arthritis
Source: Front Pharmacol. 2022 Jan 21;12:811719. doi: 10.3389/fphar.2021.811719 (PMC8814446; doi:10.3389/fphar.2021.811719)
Supplement: Supplementary file 1 [file DataSheet1.docx]

**Table S1 Genotype of *TNF-α* -308G/A interpretation criteria**

| **Genotype** | **Fluorescence** | | |
| --- | --- | --- | --- |
|  | **FAM(-A)** | **VIC(-G)** | **ROX (internal reference)** |
| GG | - | + | + |
| GA | + | + | + |
| AA | + | - | + |


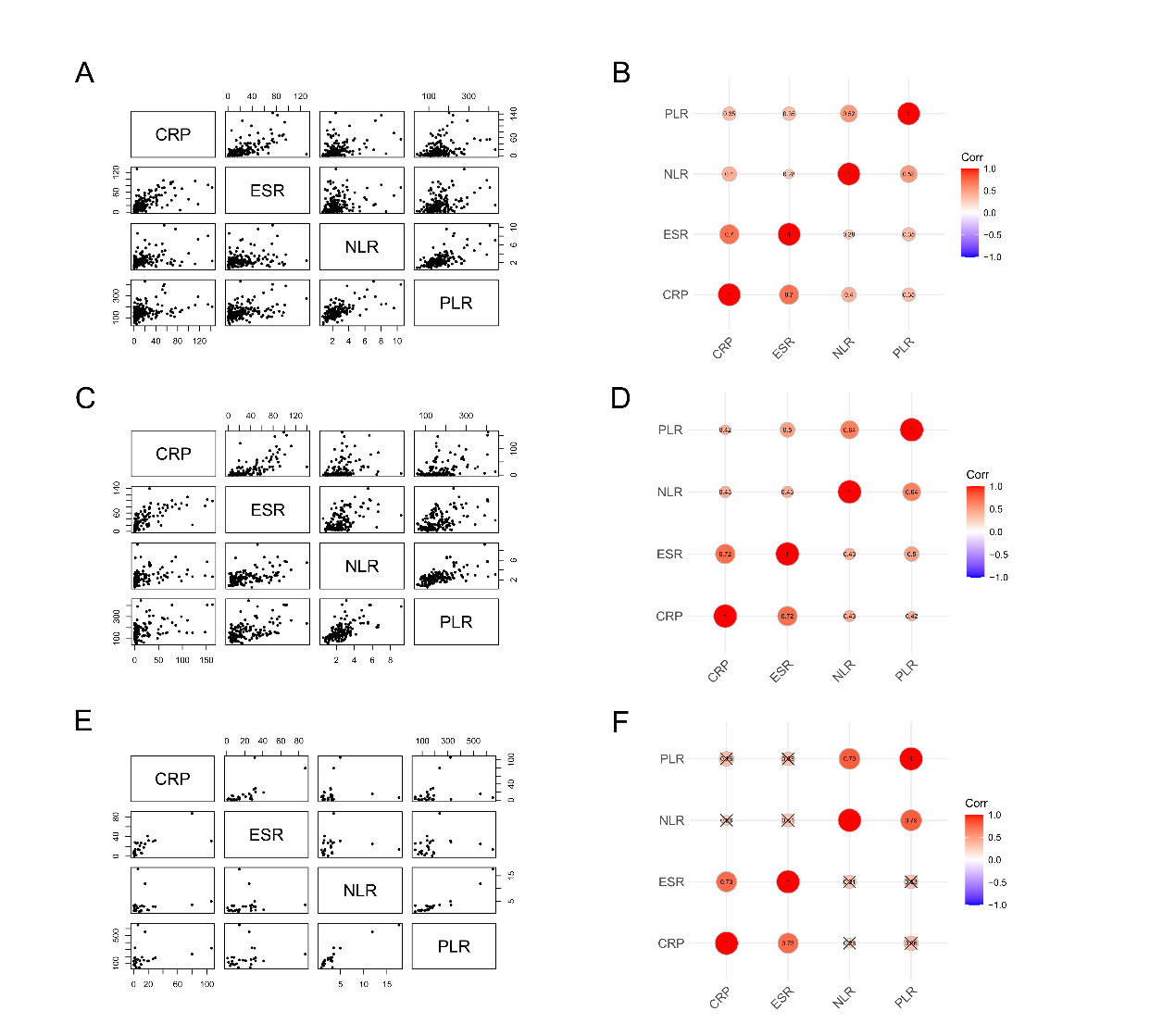


**Figure S1 Correlation analysis among CRP, ESR, NLR, and PLR**

**A-B**: correlation analysis among CRP, ESR, NLR and PLR in AS, the numbers in the circles in the right panel represented r values; **C-D**: correlation analysis among CRP, ESR, NLR and PLR in RA, the numbers in the circles in the right panel represented r values; **E-F**: correlation analysis among CRP, ESR, NLR and PLR in PsA, the numbers in the circles in the right panel represented r values. The “X” in the circles indicated *P*>0.05
